# Superconducting Quantum Magnetometers for Brain Investigations

**DOI:** 10.3390/s25154625

**Published:** 2025-07-25

**Authors:** Carmela Bonavolontà, Antonio Vettoliere, Pierpaolo Sorrentino, Carmine Granata

**Affiliations:** 1 Consiglio Nazionale delle Ricerche, Institute of Applied Sciences and Intelligent Systems, via Campi Flegrei 34, 80078 Pozzuoli, Italy; carmela.bonavolonta@cnr.it (C.B.); antonio.vettoliere@cnr.it (A.V.); ppsorrentino@gmail.com (P.S.); 2 Department of Biomedical Sciences, University of Sassari, 07100 Sassari, Italy

**Keywords:** quantum magnetometer, dc-SQUID, magnetic field noise, Josephson junctions, magnetoencephalography

## Abstract

This review article aims to provide an overview of superconducting magnetic quantum sensors and their applications in the biomedical field, particularly in the neurological field. These quantum sensors are based on superconducting quantum interference devices (SQUIDs), the operating principles of which will be presented along with the most relevant characteristics. Emphasis will be placed on the magnetic flux and magnetic field noise, which are essential for applications, especially brain investigations requiring ultra-high magnetic field sensitivity. The main configurations of SQUID magnetometers used for highly sensitive applications will be shown, stressing their design aspects. In particular, the configurations based on the superconducting flux transformer and the multiloop will be explained. We will discuss the most critical application of SQUID magnetometers, magnetoencephalography, which measures the weak magnetic signals produced by neuronal currents. Starting from the realization of a multichannel system for magnetoencephalography, we will present an accurate comparison with recent systems using optically pumped magnetometers. Finally, we will discuss the main clinical applications of magnetoencephalography.

## 1. Introduction

New quantum technologies are gaining increasing attention and will bring about a new technological revolution that will characterize the 21st century.

Although quantum computing has received the most media attention, mature and, in some ways, already-on-the-market quantum technologies include quantum communications and quantum sensors. Although less known to the general public, quantum sensors’ high sensitivity has allowed the realization of applications of considerable interest in many fields.

Concerning high-sensitivity magnetometry, the functional imaging of the brain through the measurement of the very weak magnetic fields generated by neuronal currents [1,2,3] and the investigation of the magnetic properties of matter at the nanoscale level [4,5,6,7] are of particular interest. For these applications, the most employed quantum magnetic sensors are Superconducting QUantum Interference Devices (SQUIDs) [8,9,10,11], optically pumped atom magnetometers (OPMs) [12,13], and magnetometers based on nitrogen-vacancy centers in diamond [14,15]. The above quantum sensors, in particular OPMs and SQUIDs, show sensitivities limited only by the basic principles of quantum physics. They can measure magnetic fields smaller than 10^−15^ T or magnetic moments of a few Bohr magnetons [5].

Traditional MEG systems use SQUID sensors, which are based on superconductor materials. Superconductivity is considered the most extraordinary manifestation of quantum physics at a macroscopic level. It consists of the absence of electrical resistance in some materials when they are cooled to a temperature lower than a specific temperature, which is called the critical temperature. The critical temperatures for superconducting metals are very low, less than 10 K, so they must be cooled in liquid helium (4.2 K). The total absence of electrical resistance is not the only property of superconductors: they are perfect diamagnets, which entirely expel the magnetic field from their interior (Meissner effect). The microscopic quantum theory explaining superconductivity predicts that below the critical temperature, billions upon billions of electrons instantly couple together thanks to a weak attractive interaction, forming a set of electron pairs (Cooper pairs). This so-called superconducting condensate flows through the superconductor without encountering any obstacles and, therefore, experiences no electrical resistance. Among the direct consequences of the superconductivity phenomenon are two effects that are at the basis of the operating principle of SQUIDs: the quantization of the flux inside a superconducting ring and the Josephson effect.

In this review, the second section will describe the operating principles of SQUIDs as well as the basic operation mode concerning the current-voltage characteristic as a function of applied magnetics flux, and the main magnetometer configurations will be explained. Two of the most important points of merit of SQUIDs, the power spectral density of magnetic flux and the 1/f noise, will be reported. The SQUID’s sensitivity and noise performance will be compared with that of the Optical Pumped Magnetometer (OPM), the main promising alternative to the SQUID, which will be introduced and described.

The third section is dedicated to magnetoencephalography (MEG), one of the most important biomagnetic applications of SQUID sensors. The basic principles of this noninvasive method, as well as the system components and signals, will be described, highlighting its advantages and drawbacks.

The last section is dedicated to the clinical applications of the MEG. The paragraph reports the main clinical disease in which the MEG is involved as a noninvasive diagnostic technique useful for studying the origin and diagnosis of psychiatric and neurological disorders.

## 2. SQUIDs

### 2.1. Working Principles

The SQUID is considered the most sensitive magnetic flux detector, with an energy sensitivity that approaches the quantum limit. SQUIDs come in two types: a dc-SQUID, based on the interference effects in the two-junction superconducting loop; and an rf-SQUID, which is based on the variation of the rf impedance or loss as the flux bias is changed, in a one-junction superconducting loop. Both categories of SQUIDs have been used to make measurements requiring higher magnetic field sensitivity than non-superconducting devices. Furthermore, SQUIDs can be configured for the measurement of magnetic field gradients, magnetic moment, currents, voltages, and displacements. Generally, a SQUID can measure with extreme sensitivity any physical quantity that can be transformed into a magnetic flux.

The operation principle of a SQUID is based on the Josephson effect and the flux quantization in a superconducting ring. The Josephson effect occurs in a Josephson junction, which consists of two superconductors separated by a thin insulator layer (the barrier). A DC bias current at the junction causes the voltage across it to remain zero until a current reaches a value known as the Josephson critical current, *I*_0_. The junction then switches to a resistive state, which results in a voltage appearing across it. Further details about the Josephson effect can be found in reference [16].

Regarding the flux quantization, it states that the magnetic flux threading a superconducting loop exists only in multiples of the flux quantum *Φ*_0_ (*Φ* = n*Φ*_0_ with *Φ*_0_ *= h/*(*2e*) = 2.07 × 10^−15^ T m^2^, where *h* is the Planck constant and *e* is the electron charge [17]).

In this section, attention will be focused on the description of the dc-SQUID, which consists of a superconductor loop interrupted by two Josephson junctions, preferably with similar properties.

**Figure 1:**
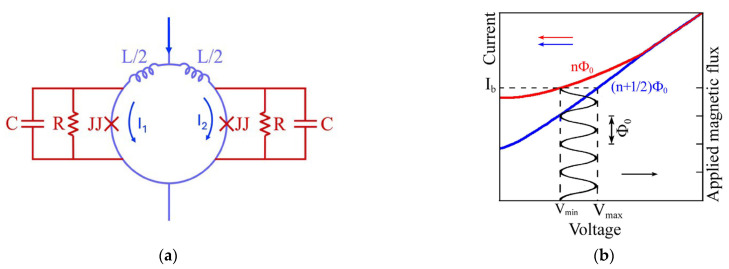
(**a**) Schematic circuit diagram of dc-SQUID; (**b**) *I*-*V* characteristics and voltage-applied magnetics flux dependence.

The typical SQUID configuration is schematically shown in Figure 1a. The two Josephson junctions are in a parallel configuration, so the critical current of the SQUID is *I_C_* = *I*_1_ + *I*_2_ (with *I*_1_ and *I*_2_ being the current in the junction 1 and 2, respectively) or *I_c_ =* 2*I*_0_, if the Josephson junctions are identical. In the presence of an external magnetic field threading the SQUID loop, *I_C_* oscillates with a period of one flux quantum. This is due to the interference of superconducting wave functions in the two arms of the SQUID and analogous to the two-slit interference in optics. In the superconducting loop, there is magnetic flux only in multiples of the flux quantum. When a change in flux occurs, currents flow to oppose it. The voltage across the SQUID is a periodic function of the external magnetic flux threading the SQUID ring with a period equal to the flux quantum *Φ*_0_.

Figure 1a shows a sketch of dc-SQUID, with the shunt resistance R and the capacitance C shown for each junction. Figure 1b reports the current–voltage characteristic of the dc-SQUID.

At first glance, the current–voltage curve looks like that of a single junction, with the total critical current being a periodic function of the magnetic flux applied to the superconductor loop. The *I*-*V* graph displays two curves corresponding to integer (n*Φ*_0_) and odd half-integer (n + 1/2) *Φ*_0_ values of the applied flux, measured in units of *Φ*_0_. Suppose the SQUID is biased by a constant current into the finite voltage regime. In that case, the time-averaged voltage across the junctions is also a periodic flux function with a periodicity of *Φ*_0_ (see Figure 1b).

Figure 1a reports the equivalent electrical circuit of a SQUID based on the resistively shunted model [18], in which the Josephson junction has a critical current *I*_0_ and is in parallel with a capacitance *C* and resistance *R* having a current noise source associated with it [19,20]. The *R* value is related to the hysteresis in the *I*-*V* characteristic of a junction or a SQUID. In particular, if the Stewart McCumber parameter _βc_ = 2π*I_C_CR*^2^/*Φ*_0_ < 1, there is no hysteresis [21].

**Figure 2:**
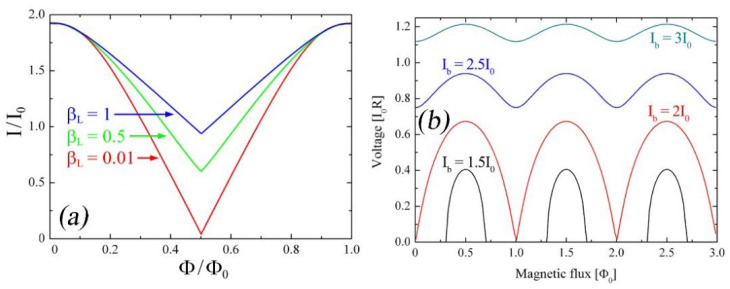
(**a**) Numerical simulation of the critical current of a SQUID as a function of the external magnetic flux threading the loop for three different *β_L_* values [5]; (**b**) voltage–magnetic flux characteristics computed for *β_L_* = 1 and *β_C_* = 0 and for *I_B_/I*_0_ = 1.5, 2.0, 2.5, and 3.0.

The flux quantization in the presence of a superconducting ring, including two Josephson junctions, can be written as follows [16]:

$$\phi_{1}-\;\phi_{1}=2\pi \frac{\Phi }{\Phi_{0}}=2\pi \frac{\Phi_{a}+L\;J}{\Phi_{0}}$$

where $\phi_{1}$ and $\phi_{2}$ are the phase differences of the superconducting wave functions across the two junctions and $\Phi_{a}+L\;I_{C}$ is the total flux threading the SQUID loop given by the external flux *Φ_a_* and the self-flux $L\;I_{C}$ produced by the screening current circulating into the SQUID loop with an inductance *L*. The circulating current can be expressed as *J* = (*I*_1_ *− I*_2_)/2. In the case of zero voltage state, applying the Kirchoff method to the circuit of Figure 1a and combining the two Josephson equations (*I = I*_0_ sin ϕ and ∂*F*/∂*t* = 2 *eV*/*ħ*) supposing that the two junctions are identical, we obtain

(1) $$I\left(\Phi \right)=I_{1}+I_{2}=I_{0}\sin\phi \cos\left(\pi \frac{\Phi }{\Phi_{0}}\right)$$

(2) $$\frac{\Phi -\Phi_{a}}{\Phi_{0}}=\frac{LJ}{\Phi_{0}}=\frac{LI_{0}(\sin\phi_{1}-\sin\phi_{2})}{\Phi_{0}}=\beta_{L}\cos\phi \sin\left(\pi \frac{\Phi }{\Phi_{0}}\right)$$

where $\phi =(\phi_{1}+\;\phi_{1})/2$ and *β_L_ = 2LI*_0_*/ϕ*_0_ is the inductance parameter. The SQUID characteristic strongly depends on the β_L_, and if the SQUID inductance is very small, β_L_ ≈ 0. Consequently, *Φ ≈ Φ_a_*, the SQUID critical current has a simple sinusoidal behavior, and the modulation depth, defined as Δ*I_C_* = *I_C_* (*Φ_a_* = 0) − *I_C_* (Φa = Φ_0_/2), is equal to 2*I*_0_, that is, the SQUID critical current modulates to zero as reported in Figure 2 (red curve).

If *β_L_* is not zero, ΔI_C_ decreases by increasing the *β_L_* value as shown in Figure 2, where *I_C_*, as a function of the external magnetic flux, is reported for three different *β_L_* values. The curves were obtained by numerically solving Equations (1) and (2). An estimation of the critical current modulation depth is given by Δ*I_C_/I_C_* = 1/(1 + *β_L_*) [22]. For *β_L_* = 1, the critical current modulates by 50%, and for *β*_L_ >> 1, Δ*I_C_/I_C_* decreases, as 1/*β_L_*.

The voltage state involves the presence of an oscillating current and voltage as predicted by the second Josephson equation (∂*F*/∂*t* = 2 *eV*/ℏ). In this case, the equations describing the SQUID dynamic are obtained by including in the Kirchhoff law the current terms due to the voltage across the resistance (V/R) and the capacitance (*CdV*/*dt*). Applying the Kirchhoff law to both SQUID arms, we obtain the following two equations.

(3) $$\frac{I_{c}}{2}+J=I_{0}\sin\phi_{1}+\frac{\Phi_{0}}{2\pi R}\frac{d\phi_{1}}{dt}+\frac{\Phi_{0}C}{2\pi }\frac{d^{2}\phi_{1}}{dt^{2}}+I_{N,1}\;\frac{I_{c}}{2}+J=I_{0}\sin\phi_{2}+\frac{\Phi_{0}}{2\pi R}\frac{d\phi_{2}}{dt}+\frac{\Phi_{0}C}{2\pi }\frac{d^{2}\phi_{2}}{dt^{2}}+I_{N,2}$$

The terms *I_N_*_,1_ and *I_N_*_,2_ are the Nyquist noise associated with the shunt resistors *R*.

The above Equation (3), together with the Equation (2), provide a complete description of the SQUID characteristics. The voltage is given by

(4) $$V=\frac{1}{2}\left[\frac{d\left(\phi_{1}\left(t\right)+\phi_{2}(t)\right)}{dt}\right]$$

The average value of *V*(*t*) allows us to calculate the current–voltage characteristic (*I*-*V*), the voltage–magnetic flux characteristic (*V*-*Φ*), and the voltage responsivity, namely the slope of the *V*-*Φ* curve in the magnetic bias point V_Φ_ = ∂*V*/∂*Φ_a_*. The power spectral density (PSD) of *V*(*t*) gives the PSD of the voltage noise (S_V_) and of the magnetic flux noise (S_Φ_ = S_V_/V^2^_Φ_). In the simplest case, where *I_N,_*_1_ = *I_N,_*_2_ = 0 and *β_L_, β_C_* << 1, Equations (2) and (3) can be easily solved, providing the following equation for the SQUID *I*-*V* characteristic:

(5) $$V\left(\Phi_{a},I\right)=\frac{R}{2}\sqrt{I^{2}-{\left(2I_{0}\cos\left(\pi \frac{\Phi_{a}}{\Phi_{0}}\right)\right)}^{2}}$$

The voltage swing or peak-to-peak modulation defined as Δ*V_S_* = *V*(*Φ*_0_*/*2) *− V*(*Φ*_0_) is given by

(6) $$\Delta V_{S}\left(I\right)=\left[\frac{RI}{2}-\frac{R}{2}\sqrt{I^{2}-{\left(2I_{0}\right)}^{2}}\right]$$

The maximum value is obtained for *I =* 2*I*_0_, that is, Δ*V_S_* = *I*_0_*R*.

In the more general case, Equations (2) and (3) are numerically solved [23,24], providing *ϕ*_1_(*t*) and *ϕ*_2_(*t*), which allow us to compute all the SQUID characteristics.

Figure 2b reports the *V*-*Φ* characteristics for three different values of the *I_B_/I*_0_ ratio and *β_L_* = 1, *β_C_* = 0 for all curves. As expected, the *V*-*Φ* curve shows a periodic dependence (with a period equal to *Φ*_0_) of the voltage across the SQUID on the external magnetic flux treading the SQUID ring. The *V*-*Φ* amplitude (*V*(0) − *V*(*Φ*_0_*/*2)) depends on the bias current and *β_L_* value and reaches its maximum for *I_B_ = 2I*_0_.

Hence, a non-hysteretic SQUID can be considered as a magnetic flux–voltage transducer and can be employed as a magnetic flux detector. In this case, Δ*Φ_a_* = Δ*V/V_Φ_*. Typically, in this configuration, the SQUID is biased with a constant current close to I_c_ and an external magnetic flux *Φ_a_ = Φ*_0_/4 to maximize the *V_Φ_*.

For a SQUID device, the important figures of merit are the power spectral density of the voltage, *S_V_*, and the spectral density of magnetic flux, $S_{\Phi }^{1/2}=S_{V}^{1/2}/V_{\Phi }$.

Solving numerically the SQUID equation for *β_L_* = 1 and with thermal noise induced by the shunt resistors, the minimum $S_{\Phi }^{1/2}$ corresponds to approximately 1.6 *I_c_*. The values of *S_V_* in the white region (independent from the frequency), *V_Φ_* and $S_{\Phi }^{1/2}$, are given [5,19,20]:

(7) $$S_{V}≅16\;k_{B}TR;\;\;V_{\Phi }\approx \frac{R}{L}\;\;\Rightarrow \;\;S_{\Phi }^{1/2}=\frac{S_{V}^{1/2}}{V_{\Phi }}≅4\;\sqrt{\frac{k_{B}\;T\;}{R}\;}L$$

To compare SQUIDs with different inductance, SQUID noise is often presented as the noise energy per bandwidth:

(8) $$\varepsilon ≅\frac{S_{\Phi }}{2\;L}≅\frac{8\;k_{B}\;T\;L}{R}$$

It is expressed in units of *ħ*. In other words, a SQUID can reach an energy resolution per bandwidth of a few *ħ*, which is limited by the uncertainty principle of quantum mechanics [25]. It has been proven that the conditions *β_L_ =* 1 and *Φ* = 0.25 *Φ*_0_ optimize SQUID performance.

It is also useful to mention another parameter that characterizes the SQUID device performance: low frequency noise (1/f or flicker noise). Investigations of this noise are important for biomagnetism, geophysics, and quantum computing. As reported in a pioneering work by Koch et al. [26], the two main sources of low-frequency noise in a dc-SQUID are known: the first is the critical current fluctuations of the Josephson junctions (critical current noise), and the second is the motion of the magnetic vortices trapped in the SQUID body (flux noise).

The magnetic flux noise due to a critical current fluctuation is caused by applying a constant current (voltage state) to the SQUID. Electrons tunnelling through the barrier may be subject to critical current fluctuations, which can trap them in a defect and release them later. The presence of a trap leads to a local change in the height of the energy barrier, affecting the critical current density in that area. The critical current I_0_ of the junction between two values is randomly switched by a single trap. It can be shown that this process results in a 1/f spectrum. Fortunately, this noise can be minimized using a suitable readout method that reverses the bias current at a frequency above the 1/f noise corner [27].

The contribution of 1/f magnetic flux noise is also due to the presence of defects in the body of the SQUID or in any superconductor circuit elements connected to it, which can act as pinning sites for a magnetic vortex during the cooling process. If the thermal energy is high enough, the vortex energy can overcome the pinning energy and can move back and forth between two or more nearby pinning sites. This change can affect the flux linked to the SQUID.

To reduce this noise, the number of defects must be decreased using a very high-quality superconducting film, or the superconducting component must be designed so that vortices cannot enter. Narrow-linewidth structures are one way to achieve this. Specifically, the linewidth should be lowered to below (*Φ*_0_*/B*)^1/2^ [28], where B is the magnetic field used to cool the device. This type of noise can be reduced by improving the pinning of the vortex inside superconducting structures, thereby preventing its motion. For this purpose, appropriate structures can be included in the SQUID ring, such as ditches and apertures [29,30]. These structures reliably trap the vortex, preventing it from moving and reducing flicker noise. The efficacy of the flux dam, a vulnerable element in the superconductor configuration, in mitigating 1/f noise has been substantiated in the context of a SQUID device undergoing movement in an ambient magnetic field [31]. In fact, the flux dam limits circulating supercurrents, which induce vortex generation.

**Figure 3:**
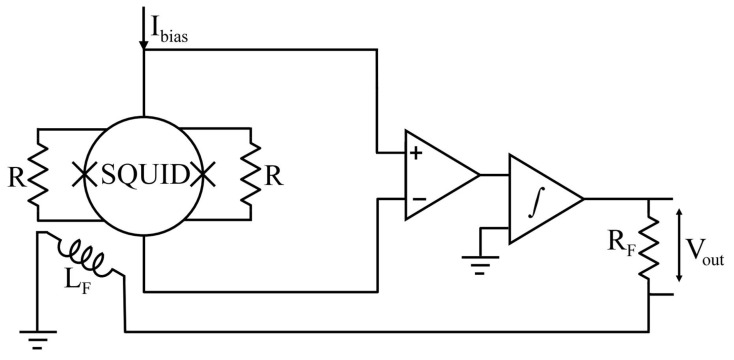
Flux locked loop for dc-SQUID used to enhance the linear dynamic range of the device. The output SQUID signal, after integration, is fed back as a current through a feedback resistor R_F_ to a coil inductively coupled (L_F_) to the SQUID.

Moreover, the *V*-*Φ* curve is linearized through SQUIDs operating in a flux-locked loop configuration, in which the voltage changes across the SQUID, induced by an applied flux, are amplified and fed back as an opposing flux. The feedback circuit’s functions include linearizing the response of the SQUID, providing a straightforward means of measuring the intrinsic noise of the SQUID, and enabling one to track inputs equivalent to many flux quanta. The input stage of the electronic circuitry has been designed so that only a negligible amount of noise is added to the intrinsic noise of the SQUID. Figure 3 [32] shows the widely used scheme.

The flux fed back in opposition to the applied flux enables the flux in the SQUID to remain constant; the voltage developed across R_F_ is proportional to the applied flux. Measuring the intrinsic flux noise of the SQUID is possible in the absence of any input signal by connecting the output voltage to a spectrum analyzer.

For unshielded applications, the slew rate (maximum rate of change of flux) is a more important figure of merit. For an ideal single-pole integrator, the slew rate is 2πf_1_*Φ*_0_/4, where f_1_ is the frequency at which the open-loop gain of the feedback loop falls to unity [33]. A two-pole integrator can improve the slew rate at low frequencies [34].

### 2.2. Superconducting Magnetometer and Gradiometer Configurations

**Figure 4:**
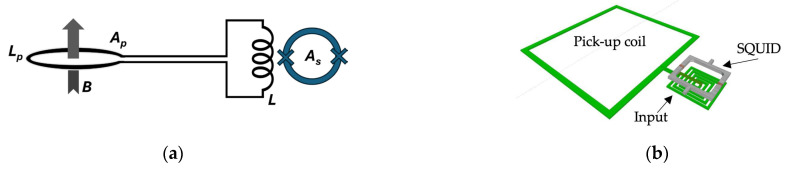
(**a**,**b**) The transformer used to enhance the SQUID sensitivity, shown in a schematic representation. The sensor’s effective area is increased by the super-conducting flux transformer, which is inductively coupled to the SQUID loop.

SQUIDs are extremely sensitive to magnetic flux, but their small size makes them less suitable for measuring tiny magnetic fields. Increasing the SQUID loop area increases the magnetic field sensitivity. The magnetic field sensitivity cannot be enhanced by expanding the geometrical area of the SQUID ring because the flux noise rises with the ring inductance. This problem can be avoided by using an additional superconductor (a flux transformer) in conjunction with the SQUID, thus increasing its sensitivity to magnetic fields. It consists of a primary coil working as a magnetic flux pick-up (pick-up coil), connected in series with a secondary coil magnetically coupled to the SQUID (input coil). The magnetic flux is particularly coupled to the low-inductance SQUID loop from a larger external pick-up coil. The configuration of such a transformer is shown schematically in Figure 4a.

The magnetic-field noise is $S_{B}^{\frac{1}{2}}\left(f\right)=\frac{S_{\Phi }^{\frac{1}{2}}\left(f\right)}{A_{eff}}$, where A_eff_ is the effective area of the magnetometer. Clearly, one wants to make A_eff_ as large as possible without increasing *S_Φ_*(*f*), to produce high sensitivity to magnetic fields. It is worth nothing that the effective area is smaller with respect the geometrical area of the pick-up coil.

The SQUID magnetometer design [35,36,37] shown in Figure 4 consists of a large pick-up loop of inductance *L_p_* and area *A_p_* inductively coupled to the SQUID body of inductance, L_i_ << L_p_. When a magnetic field *B* is applied, a screening current *I* = *B A_p_/*(*L_p_ + L_i_*) is induced in the pick-up loop, which in turn links a flux *Φ_S_* to the SQUID.

(9) $$\Phi =\frac{M_{i}}{L_{i}+L_{P}}\Phi_{P}=\frac{k_{i}\sqrt{LL_{i}}}{L_{i}+L_{P}}\Phi_{P}$$

where *M_i_* represents the mutual inductance between the SQUID loop and the input coil, *L_i_* and *L_p_* represent the inductances of the input and pick-up coils, respectively, and *k_i_* is a coupling factor. It is possible to obtain the spectral density of the magnetic field noise *S_B_*^1/2^ of the SQUID magnetometer [23]:

(10) $$S_{B}^{\frac{1}{2}}=\frac{S_{\Phi ,p}^{\frac{1}{2}}}{A_{P}}=\frac{L_{i}+L_{P}}{M_{i}A_{P}}S_{\Phi }^{\frac{1}{2}}=B_{\Phi }S_{\Phi }^{\frac{1}{2}}\;\;$$

(11) $$B_{\Phi }=\frac{L_{i}+L_{P}}{M_{i}A_{P}}$$

where *B_Φ_* is the magnetic flux to magnetic field conversion efficiency or SQUID magnetometer sensitivity. This is a fundamental parameter for a SQUID magnetometer and assumes the minimum value when *L_p_ = L_ị_*. The effective area A_eff_ in this configuration is given by 1/*B_Φ_*. Hence, the magnetometer design can be optimized by minimizing the value of *B_Φ_* in Equation (11). If the flux transformer is integrated, an excellent coupling to the SQUID is obtained using a Ketchen-type design [38].

**Figure 5:**
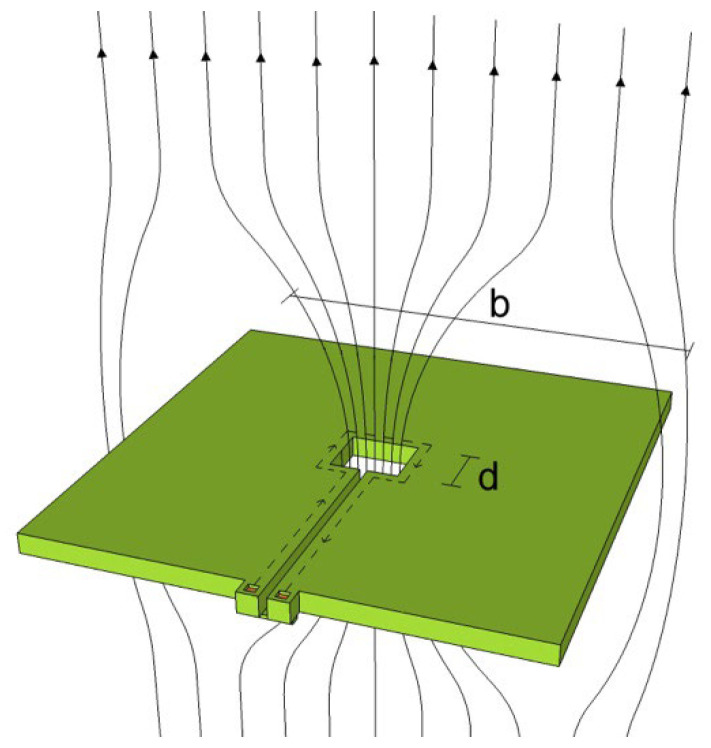
Scheme of a dc-SQUID in a washer configuration. Due to flux focusing effect, this configuration allows increasing the effective area with respect to the hole geometrical area.

The SQUID loop, in a square washer configuration (Figure 5), is magnetically coupled to a multiturn thin-film input coil connected to a square single-turn pick-up loop.

In the washer configuration, SQUID inductance depends only on the hole size; it does not depend on the outer washer dimensions, as reported by numerical simulation in [39]. The coupling between the washer and the input coil is efficient, and the input coil’s inductance depends on the number of turns and the hole’s inductance. The required number of input coil turns can be accommodated by varying the outer washer dimensions to match a suitable load, thereby adjusting the input coil inductance. The Josephson junctions have been placed on the outer edge of the square loop, away from the higher field region at the center of the square hole.

The use of a slit through the conductor loop is necessary, but this introduces parasitic inductances. The coupling efficiency is reduced because these are only partially coupled to the coil turns. Therefore, it is preferable to avoid very long slits.

The washer structure focuses flux into the central hole (flux focusing effect), making the effective area of the dc-SQUID greater than the geometrical one (Figure 5). For a square washer, it can be demonstrated that the effective area is *A_eff_* = *k b d*, where *k* is a numerical constant close to unity, under the hypothesis that the washer width *b* is larger than the hole dimension *h*. The ratio of the effective area to the geometric area is *b*/*d* [38,40]. The flux-focusing effect is employed to fabricate SQUID sensors for several applications. Typically, magnetic flux noise is as low as 1 fT/√Hz for a SQUID magnetometer with a square pick-up coil having a length of about 1 cm.

**Figure 6:**
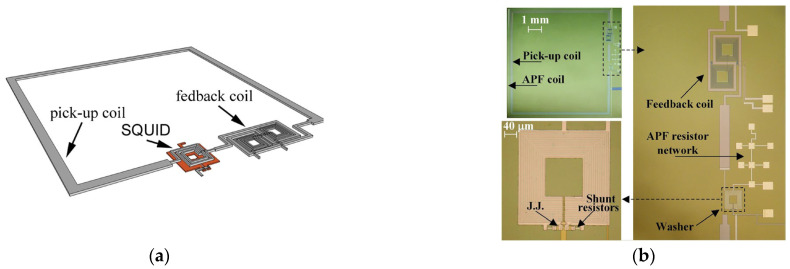
(**a**) Typical configuration of inductive coupling magnetometer in the “Ketchen scheme”; (**b**) SQUID magnetometer with APF circuit integrated on the same chip. Adapted from [11]. The pick-up coil area is 64 mm^2^.

To improve the inductive coupling between the SQUID and the input coil, some groups have put them on the same chip, which means there is less space between them and the insulating layer. In addition, an Addition Positive Feedback (APF) circuit could be integrated on the same chip to reduce the equivalent preamplifier flux noise with respect to the SQUID noise in the case of a direct-coupled readout scheme. This makes the voltage–magnetic flux characteristics (*V*-*Φ*) asymmetric, so if the SQUID is biased on the steeper side, an effective increase of the flux-to-voltage transfer coefficient is achieved (V_Φ_ = ∂*V*/∂*Φ*). The integrated APF circuit includes a resistive network to adjust the gain of the APF for optimum operation of the device. Crosstalk between neighboring sensors can be reduced by the bipolar design of the feedback coil, which consists of two multiturn coils [41]. In Figure 6, a fully integrated magnetometer based on superconductive flux transformer is reported. The B_Φ_ of this magnetometer is 0.7 nT/*Φ*_0_, corresponding to an effective area of 2.85 mm^2^, while the magnetic flux noise is 1.5 fT/Hz^1/2^.

The design and size of the superconducting flux transformer are fundamental for SQUID magnetometers based on Ketchen-type design. Increasing the size of the pick-up coil increases the sensitivity, that is, the B_Φ_ conversion factor decreases. However, as specified above, to optimize the B_Φ_ transfer factor, the value of the input coil inductance must be equal to the value of the pick-up coil inductance. Therefore, if the size of the pick-up coil is increased, in principle, the number of turns of the input coil must also be increased to increase its inductance to match the inductance of the pick-up coil. In any case, it is preferable not to exceed a pick-up coil area greater than 50–70 mm^2^ in order not to lose spatial resolution. If it is possible to have sensors that are not very large without sacrificing sensitivity in the magnetic field, then it will be possible to create multisensory helmets that contain a greater number of SQUIDs that are closer together. This leads to an increase in spatial resolution, which is fundamental for the reconstruction of neuronal sources in the case of brain investigation by magnetoencephalography.

In this regard, an effective way to increase the magnetic field sensitivity of a SQUID device while maintaining a small value of the pick-up coil area (10–30 mm^2^) is to increase the inductance between the input coil and the SQUID washer *M_i_*, increasing the washer hole. Obviously, this involves an inevitable increase in the SQUID inductance L and a consequent increase in the noise parameter *β_L_*. In this case, it has been demonstrated that the insertion of an appropriate resistance in parallel to the SQUID avoids the degradation of the performance in terms of magnetic flux noise [41,42,43,44].

It is important to stress that it is not necessary to have a magnetic field noise lower than 1 fT/Hz^1/2^, as this is the limit of most of the shielded rooms used in multichannel systems for magnetoencephalography. It is quite useful to have stable magnetometers with low noise at low frequency. In fact, even in commercial systems for magnetencephalography, the noise in the magnetic field is of the order of 1–2 fT/Hz^1/2^, with a low frequency corner of a few Hz, and the size of the pick-up coil is below 100 mm^2^.

An alternative multilayer approach to achieving large effective areas is the multiloop SQUID magnetometer originally proposed and demonstrated by Zimmerman [45] with a machined niobium device. The essential idea is to connect N loops in parallel, thus reducing the total inductance to a level acceptable for a SQUID while keeping the effective area large.

**Figure 7:**
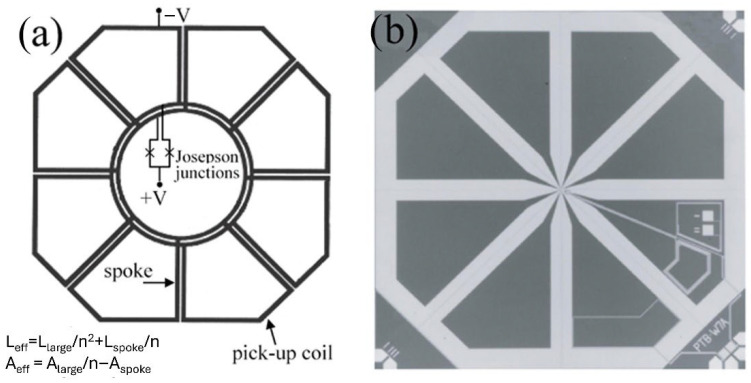
Schematic diagram (**a**) and picture (**b**) of a multiloop magnetometer. Here, n = 8 is the number of pick-up loops connected in parallel, reducing the SQUID inductance, L_large_ and A_large_ are the inductance and the area of the large octagonal loop, while A_spoke_ and L_spoke_ represent the average area and average inductance of one “spoke” line. Adapted from [51].

Sensitive multiloop SQUID magnetometers were developed by Drung et al. [46,47] (Figure 7) based on their niobium thin-film technology. In the thin-film multiloop magnetometer, shown schematically in Figure 7a, N loops are connected in parallel with the connection made at the center via coplanar lines. With an outer diameter of 7.2 mm and eight parallel loops, this magnetometer exhibits a B_Φ_ = 0.45 nT/Φ_0_ corresponding to an effective area of 4.5 mm^2^ and magnetic field noise of about 1 fT/√Hz, down to a few Hz at 4.2 K. These devices have been successfully used in multichannel biomagnetic systems [48,49,50].

The two junctions connect the central trilayer’s upper and lower superconducting films. A multiloop magnetometer is more advantageous than a flux-transformer coupled magnetometer since the current induced in each N loop when rotated in the Earth’s magnetic field is much smaller than that induced in a single loop of the same area. Additionally, the device lacks closed superconducting loops, which means the maximum induced supercurrent is restricted to the critical current of the junctions.

**Figure 8:**
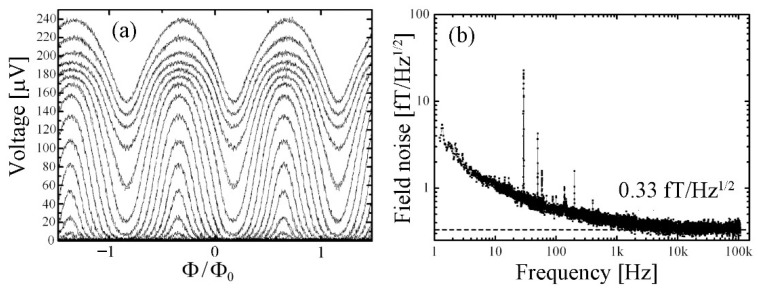
(**a**) V-Φ characteristics for different bias currents and (**b**) magnetic field noise spectrum of a multiloop SQUID magnetometer measured at liquid helium temperature (4.2 K) (reproduced with permission of Elseiver publishing) [53].

A multiloop magnetometer based on low-capacitance cross-type Josephson submicron junctions exhibited a magnetic field noise as low as 0.33 fT/√Hz (Figure 8) [52].

**Figure 9:**
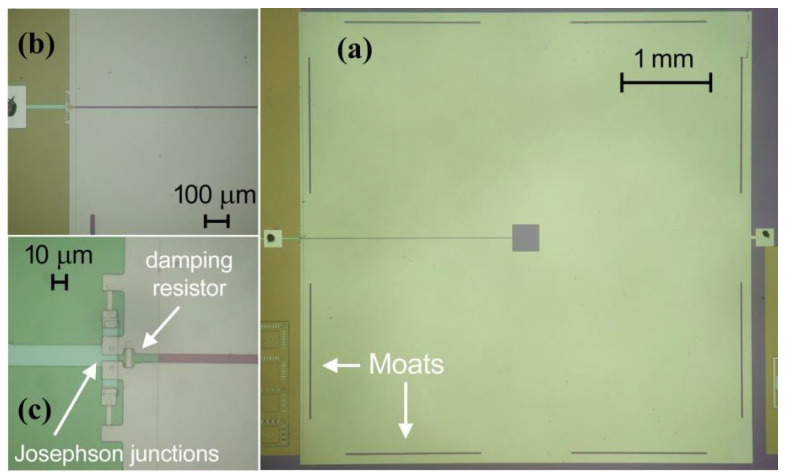
Pictures of dc-SQUID magnetometer. (**a**) The large washer and the moats near the edge to trap magnetic vortices are visible. (**b**,**c**) Two details showing the Josephson junctions, the shunt resistors, and the damping resistor. Adapted from [42], an open-access article distributed under the terms of the Creative Commons Attribution License (CC BY).

Recently, a new dc-SQUID magnetometer with an alternative design to the typical ones, suitable for high-sensitivity applications, has been suggested (Figure 9).

The design of the dc-SQUID magnetometer consists simply of a large washer-shaped loop that is interrupted by two Josephson junctions. A single coil made of a square superconductor structure, including a small hole with respect to the outer dimensions, makes up the dc-SQUID ring. Meissner’s effect causes the magnetic flux lines to be focused inside the central hole.

The basic idea was to use the flux focusing effect. This was to be performed on a washer-shaped dc-SQUID. The size of the sensor was about (5 × 5) mm^2^. This was to allow high sensitivity magnetometry applications. The sensor’s performance was prevented from being degraded. This was done by ensuring the SQUID inductance was suitably damped. The simulations show that if the ratio between the shunt resistor (Rs) and the damping resistor (Rd) γ = Rs/Rd = 1, the V-Φ characteristic and V_Φ_ are almost independent of the *β_L_* value [43]. As regards the noise characteristics, in the case of a large βL value, the damping resistor seems to improve them considerably. In particular, the spectral density of the magnetic flux noise of a dc-SQUID with a *β_L_* = 10 is only about four times larger than *β_L_* = 1, while in the absence of damping (γ = 0), the magnetic flux noise is over twenty times greater [44].

This was also ensured by choosing appropriate fabrication parameters. Fabrication is less critical since there are no additional circuits, such as the superconductor flux transformer. A bare dc-SQUID with a magnetic field sensitivity of less than 8 fT/√Hz and a low-frequency noise knee of less than 2 Hz can be obtained, as shown by the characterization data [42]. The design’s simplicity means that a high-performance quantum sensor of exceptional robustness, stability, and reliability is produced.

Typically, SQUID magnetometers are not optimized for low-energy noise. However, in the case of the magnetometers shown in Figure 6 and Figure 9, considering a SQUID inductance value of 260 pH and 1180 pH, a S_Φ_^1/2^ value of 2.8 and 9.8 μΦ_0_/Hz^1/2^, respectively, and using the expression (5), an *ε* value of about 100 *h* and 240 *h* is obtained, respectively. Energy resolution per bandwidth values close to *h* are obtained with SQUIDs having a very small inductance, such as in the case of nanoSQUIDs, where it is not difficult to reach values of a few 1–2 *h*, also at T = 4.2 K [54].

As mentioned above, the sensitivity of a SQUID magnetometer based on the superconducting flux transformer not only depends on the pick-up coil size but also on the hole size of the SQUID washer and the number of turns of the input coil; thus, comparison between different sensors normalized to the area could be a bit misleading. However, we can try to compare the sensitivity of the SQUID magnetometer based on the flux transformer, multiloop, and single-washer configurations. In the three cases reported in this paper (Figure 6, Figure 7 and Figure 9), the best sensitivity is obtained with the multiloop and the flux transformer configurations, followed by the single washer design. In fact, in the case of a single washer, it is not possible to achieve sensitivities of 1–2 fT/Hz^1/2^, as an even larger washer would have to be used with an avoidable degradation of performance in terms of V-Φ characteristics, V_Φ_ transfer factor and, consequently, magnetic flux noise.

To operate in a low- or moderate-shielded room, hardware planar or axial gradiometers, or electronic gradiometers, are typically used [55]. First-order planar SQUID gradiometers with long baselines offer several advantages. Thanks to the precision of the photolithographic techniques used in their manufacture, this type of gradiometer can be produced with a higher intrinsic balance than wire-wound axial gradiometers [56,57].

**Figure 10:**
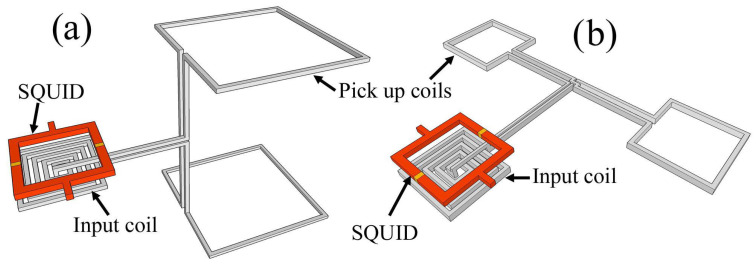
Scheme of an axial (**a**) and a planar gradiometer configuration (**b**). Adapted from [11].

The two gradiometer configurations are shown in Figure 10. The axial configuration is used to evaluate the magnetic field gradient in the z direction, while the planar configuration helps detect the x or y magnetic field gradient. In both cases, a magnetic field that is spatially uniform will induce two shielding currents of the same amplitude but in opposite directions in the coils. As a result, no current will flow into the input coil, and no magnetic flux will traverse the SQUID. If the magnetic field is not uniform, the net current circulating in the input coil is non-zero, and this couples a magnetic flux into the SQUID via the mutual inductance M_i_; in the planar case [58],

(12) $$\Phi_{S}=M_{i}J_{S}=\frac{2d^{3}M_{i}}{L_{i}+{2L}_{P}}\left(\frac{\partial B_{z}}{\partial x}\right)$$

where *d* is the distance between the pick-up coil centers (baseline). Therefore, considering Φ_n_ the SQUID magnetic flux noise, the noise gradient is given by

(13) $${\frac{\partial B_{z}}{\partial x}|}_{n}=\frac{L_{i}+{2L}_{P}}{M_{i}}=\frac{\Phi_{n}}{2d^{3}}$$

In addition, the inductance of the pick-up loop of a planar gradiometer can be matched to that of the input coil. This ensures good signal coupling with the SQUID and avoids the need for unreliable superconducting solders.

**Figure 11:**
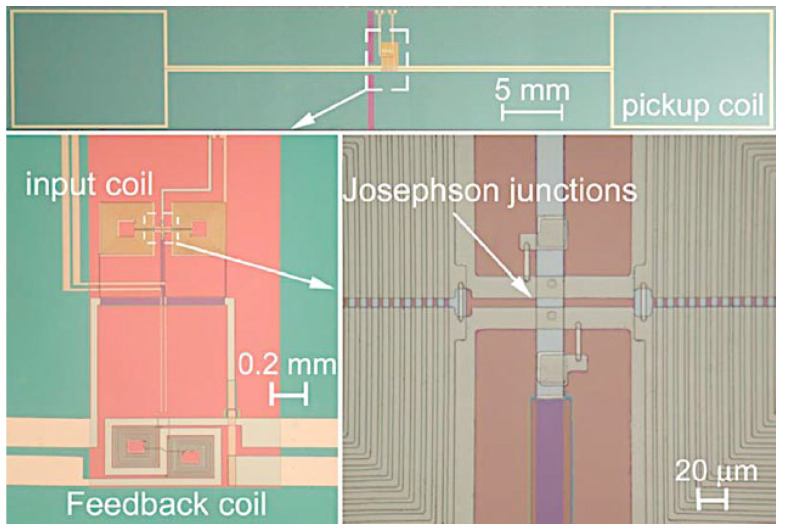
Picture of the integrated planar SQUID gradiometer, the bipolar feedback coil, which has the relative pick-up coil arranged next to it, and the SQUID, which is shaped like a washer and includes Josephson junctions, shunts, and damping resistors. Adapted from [60].

Figure 11 depicts a fully integrated SQUID gradiometer with a long baseline (50 mm) [59,60]. Thanks to the long baseline, such sensors also have adequate sensitivity for sources located in depth. Without losing both localization accuracy and sensitivity, measurement of the two tangential components simultaneously reduces the area of sensor coverage required to obtain the essential magnetic field distribution [61].

From a commercial point of view, the Ketchen-type configuration based on the superconducting flux transformer is used by the companies Starcryoelettronics and Magnicon, whose magnetometers exhibit a magnetic field sensitivity of 3 fT/Hz^1/2^ and 1.5 fT/Hz^1/2^, respectively [62,63]. On the other hand, the multiloop configuration is used by the company Supracon, which manufactures magnetometers with a magnetic field sensitivity of 3.5 fT/Hz^1/2^ [64].

Finally, it is worth mentioning other superconducting magnetometers. In 2004, hybrid magnetometers that combined a high-temperature superconducting flux transformer with a low-noise giant magnetoresistive sensor, were developed. These sensors, characterized at liquid helium temperature (4.2 K), exhibited a magnetic field sensitivity of approximately 30 fT/Hz^1/2^ [65].

A similar magnetic field sensitivity was achieved exploiting the nonlinearity of kinetic inductance of a superconductor. This kinetic inductance magnetometer contained a superconducting loop based on a single niobium nitride (NbN) thin-film layer, simplifying the fabrication processes compared with other magnetometer technologies considerably [66].

Furthermore, a superconducting magnetometer based on the long Josephson junction was developed [67]. By using a suitable array of the long Josephson junction, it is possible to reach a sensitivity less than 10 fT/Hz^1/2^. Here, the unidirectional motion of a train of flux quanta, also called flux-flow, can be exploited to sense a magnetic field, provided there is a good magnetic coupling between the pick-up loop and the long junction.

## 3. SQUID Magnetometer and Optically Pumping Magnetometer (OPM) Comparison

In recent years, the performance of optically pumped atomic magnetometers has improved significantly, becoming comparable to that of SQUID magnetometers. It is therefore interesting to briefly describe their operating principle and compare them with superconducting magnetometers.

The simplest structure of the OPM consists of a few components: a polarized light source (laser), a high-pressure vapor cell (dimension of 3–4 mm for each side), and a detection system such as a photodiode, contained in a shape of approximately 15 × 20 mm.

The core of the OPM working principle is based on the light-atom interaction that takes place in the vapor cell of optically pumped atoms, typically ^87^Rb, electrically heated at a temperature of ~150 °C to achieve optimum ^87^Rb vapor density (10^14^ cm^−3^) [68,69]. In physics terms, the Rb atom possesses a spin and, consequently, has a magnetic moment. In the zero external magnetic field, the magnetic moments of the Rb atoms are aligned arbitrarily. However, the introduction of circularly polarized laser light (795 nm), resonant with the Rubidium *D*1 transition, triggers the optical pumping process. During the optical pumping process, there are two effects to be considered: the effects of the laser and the effect of the atom spontaneously emitting energy (at 795 nm). The “selection rules” govern the two effects. The laser will always provide an increment in m_f_ (the component of the atomic angular momentum along the laser axis) and, if possible, a D1 transition (if m_f_ < 2), while if the sample is in the L = 1 state, it may spontaneously emit light (at 795 nm), reversing the D1 transition (also without any change of m_f_). The action of the laser initially increases the probability of the L = 1 state being occupied (due to D1 transitions) but also increases the probability of the L = 0, m_f_ = 2 state (highest Zeeman sub-level) occurring due to optical pumping. As atoms become trapped in the L = 0 and m_f_ = 2 state, the probability of D1 transitions drops towards 0, thus rendering the vapor transparent [12]. Therefore, if the atom already exists in the L = 0 and m_f_ = 2 state, the light transfers no energy to the sample and simply passes through the vapor, which has become transparent to the laser light (transmission of laser light to the photodiode is maximized). Once the laser optically pumps the atoms into this transparent steady state, the vapor becomes highly polarized, and the laser field transfers its angular momentum onto the alkali atoms, increasing the value of the total atomic angular momentum along the laser axis (m_f_). In this condition, many atoms occupy the same state and, hence, collectively produce a strong net magnetization (magnetic moment per unit volume), which is aligned along the axis of the laser beam [70].

The induced polarization is highly sensitive to the ambient magnetic field, with any component of the field that is perpendicular to the laser beam producing a torque on the net magnetization.

**Figure 12:**
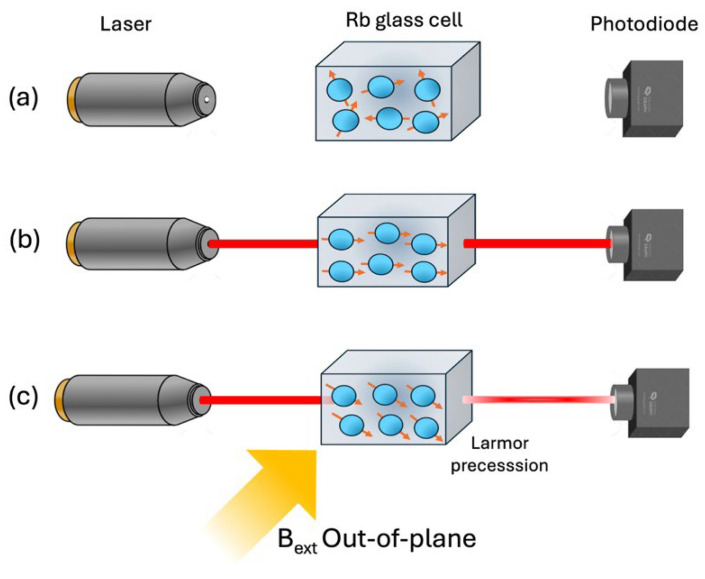
Schematic diagram of an optically pumped magnetometer (OPM) based on Rb vapor cell. (**a**) When pumping laser is off, the vapor cell is in a thermal random mixture of spin state; (**b**) polarized pumping laser light induces transition of most atoms into the same spin state, and the photodiode measures the light intensity proportional to the absorption and/or precession; (**c**) the presence of an external magnetic field B_ext_ causes the Larmor precession of the atoms in the vapor; then, the photodiode detects light passing through the vapor, the amount of which is a function of the external magnetic field.

In the presence of a magnetic field (B_ext_), the individual magnetic moments of the atoms will precess around the field direction at the Larmor frequency (ω = γ B_ext_ where γ is the gyromagnetic ratio of the atom). However, a magnetic field perpendicular to the beam causes Larmor precession, rotating the magnetic moments away from alignment, causing the magnetization vector of the vapor atoms to have a constant angle relative to the laser axis, approximately proportional to B_ext_. This causes a Faraday rotation effect, which could be detected by monitoring the intensity or polarization of the transmitted probe light, i.e., photodetector senses this change in transparency and produces an electric current proportional to the light transmitted through the vapor cell [71]. A schematic diagram of the OPM working principles is given in Figure 12.

The sensitivity of the OPMs relies on the coherence between the alkali-metal atoms. In the magnetized state of the vapor, the precessing atoms must remain in phase, but several processes, collisions with the cell wall, spin-exchange collisions, and magnetic field inhomogeneities can cause a phase difference. In other words, the atomic collisions cause a loss in coherence between atoms, and consequently, the spin alignment is degraded. Under some conditions (zero magnetic field) it is possible to suppress the decoherence caused by spin-exchange collisions, which is the dominant collision process in dense ensembles of alkali-metal atoms. Then, it is necessary to enhance the density (number of atoms per unit volume) of the rubidium atoms in the sensor (which leads to frequent collisions). The density is increased by heating the cell; in this way, the vapor pressure and the number density of the atoms in the vapor phase increase. Collisions occur so fast that coherence is not lost, and spins still process coherently, a scenario referred to as the spin-exchange relaxation-free (SERF) regime [72].

In OPM magnetometer configurations, the pumping and probing are performed by the same beam or by two different beams, with the pump beam being circularly polarized and the probe beam being linearly polarized. As mentioned above, the two most common detection modes are monitoring of the intensity or the polarization of the transmitted probe light. The polarization method has certain intrinsic advantages, such as its ability to detect very small polarization-rotation angles and a reduced sensitivity to the laser-intensity noise.

Moreover, the external magnetic field detection, i.e., arising from brain activity, can be measured adding a second oscillating magnetic field, called the ‘modulation field’, using the onboard sensor coils. This field varies at a frequency of about 1 kHz, much greater than the relaxation rate of the vapor (typically about 100 Hz). In this way, the polarization is amplitude-modulated at a frequency outside the bandwidth of the sensor [12,73]. The change in polarization is detected using a photodiode with an output voltage, demodulated using a lock-in amplifier triggered at the modulation frequency.

The polarization profile is non-linear across various magnetic fields, but it is an approximately linear function only in a restricted magnetic field range of a few nT. Moreover, the OPM can simultaneously detect components of the magnetic field vector along multiple directions [74], including radial and tangential components of the neuromagnetic field. Even if the tangential components are smaller than the radial components [75,76], they still contain useful information. Studies have shown that adding tangential components can help differentiate fields from those originating outside the head (interference) and when sensor numbers are limited [77,78].

**Figure 13:**
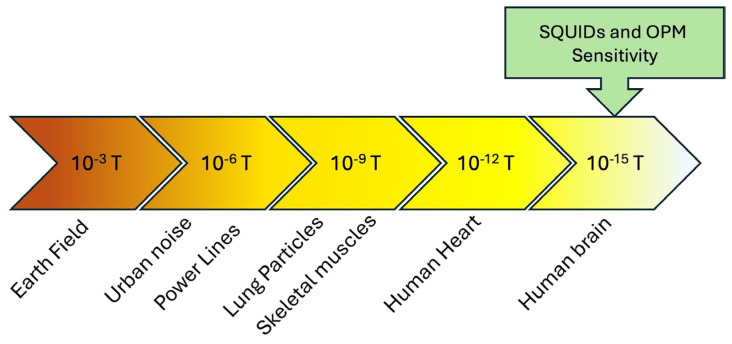
Magnetic field intensity due to the environment and human activity. SQUIDs and OPM sensors have a very low sensitivity for detection of the human magnetic field, in the order of femtoTesla.

OPMs and SQUIDs offer high sensitivity for detecting magnetic fields (Figure 13) but operate on different principles and require different temperatures. OPMs are more portable and user-friendly due to their room-temperature operation, in contrast to SQUIDs, which require cryogenic environments (usually 4.2 K), necessitating complex cooling systems and specialized electronics, which can limit portability. OPMs can reach a field sensitivity less than 1 fT/Hz^1/2^ [13]; however, commercial OPMs operate with a sensitivity in the order of 7–10 fT/√Hz, while SQUIDs are the most sensitive magnetometers available, measuring magnetic fields with a sensitivity of 1–5 fT/√Hz in the white noise frequency range [79,80,81]. OPMs operate at room temperature, making them simpler to use in a broader range of applications.

Regarding the spatial resolution, there are comparable results between the SQUIDs and OPMs. The latter, thanks to the shorter distance between the sensor’s sensitive element and the source (5–6 mm), can achieve a signal-to-noise ratio no worse than for SQUIDs. Multichannel magnetometer systems have shown a spatial resolution of magnetic activity sources comparable to SQUID systems, with fewer sensors.

Moreover, one important advantage of the OPM with respect to the SQUID is the possibility for multiaxial recording. Some new commercial OPM sensors that feature three sensing directions could improve the performance of beamformers. The linear dynamic range of the OPM is about ±5 nT, which is more limited than the ±20 nT of the SQUID [81].

## 4. Brain Investigation by Magnetoencephalography

### 4.1. What Is Magnetoencephalography

Magnetoencephalography (MEG) is one of the most important applications of SQUIDs and OPM magnetometers. MEG is a noninvasive method that allows real-time brain function imaging [81]. The technique is based on measuring magnetic fields outside the head that are generated by neuronal activity. The brain’s ionic currents produce electric and magnetic fields that can be measured on and around the scalp. Utilizing these fields allows determination of which parts of the brain are activated.

The subsequent mathematical modeling of these fields enables the generation of 3D images (termed source localization), providing a comprehensive analysis of the dynamic changes in electrical activity within the brain, including its response to diverse experimental scenarios or cognitive stimuli. The investigation encompasses a range of methodologies and analysis techniques to gain deeper insights into how electrical activity evolves in response to external triggers or internal cognitive processes.

MEG has a millisecond temporal resolution and a spatial resolution of about 2–5 mm [82]. This unique combination of temporal and spatial resolution gives MEG many advantages over other functional imaging techniques, such as functional magnetic resonance imaging (fMRI), which is limited to hemodynamic metrics and has poor temporal resolution, and electroencephalography (EEG), which has poor spatial resolution due to distortions in electrical potential caused by the skull and scalp.

The most important feature essential for MEG applications is represented by a sufficient sensitivity to measure very small magnetic fields, about 100 fT, generated by brain activity. Therefore, the conventional MEG systems are based on SQUID magnetometers [2,83]. More recently, the emerging technology of the optically pumped magnetometer has offered an alternative to SQUID sensors for the MEG system. The subject of electromagnetic activity in the human brain has a long history. The first documentation of the electrical activity of the human brain (electroencephalography, or EEG) was reported in 1929 [84], and its magnetic counterpart (magnetoencephalography, or MEG) was first measured in 1968 using room-temperature coils [83]. The low sensitivity of these early MEG measurements was significantly enhanced with the introduction of SQUID sensors, which were initially employed for MEG detection in 1972 [84].

Following this pioneering work, the field of MEG developed through several phases. Initially, single-channel devices were utilized, followed by the introduction of somewhat larger systems with five to seven channels in the mid-1980s. Subsequently, systems with approximately 20 to 40 sensors emerged in the late 1980s and early 1990s. Ultimately, the first helmet whole-cortex MEG systems were introduced in 1992. Present MEG systems possess hundreds of channels, arranged in a helmet configuration, and operate in either a seated or supine position.

Currently, MEG represents the most significant biomagnetism application, and its technology has rapidly developed in the commercial sector. This has resulted in complex systems comprising numerous channels that cover the entire cortical surface.

Some suppliers of commercial MEG systems globally include Elekta (306 sensors) [85] and CTF MEG (275 sensors) [86]. Additionally, non-commercial SQUID magnetometers with many channels were constructed in several laboratories around the world, employing both low- and high-temperature superconducting materials. As a preliminary point, it should be noted that the following examples, which are not intended to be exhaustive, can be found in [87,88,89,90,91,92,93,94,95,96,97,98].

**Figure 14:**
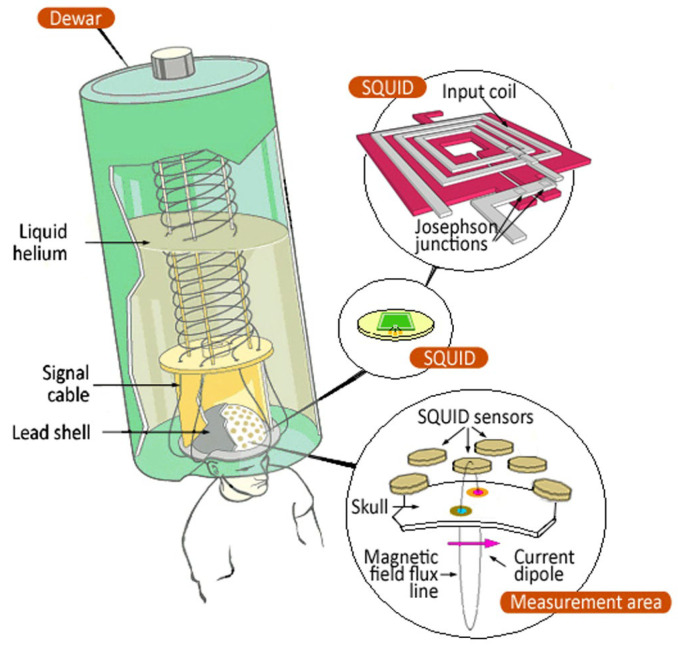
Schematic representation of a system for magnetoencephalography based on SQUID magnetometers. Adapted from [11].

Introducing helmet-type MEG systems has led to a dramatic increase in the use of SQUID sensors. The commercially available MEG instruments are based mainly on low-T_c_ SQUIDs because the high-T_c_ SQUIDs are less sensitive than their low-T counterparts and require better shielding. Figure 14 presents a SQUID-MEG illustration.

As mentioned above, the MEG measures magnetic fields on the scalp surface. Nevertheless, the current distribution of the brain that is responsible for the observed fields is typically more intriguing to the user. It is important to note that the inversion problem (i.e., calculating the current distribution from the measured magnetic field) is non-unique and ill-posed. Consequently, MEG data must be supported by additional information, physiological constraints, mathematical models, and simplifications. Alternative measuring modalities, such as electroencephalography (EEG), may be used to provide supplemental data to facilitate field inversion [99].

It has been established that both MEG and EEG measure the same sources of neuronal activity, and their information is complementary and additive [100]. Both magnetic encephalography (MEG) and electroencephalography (EEG) have excellent temporal resolution and provide functional information, which is typically combined with anatomical images obtained from magnetic resonance imaging (MRI) [101,102,103] or computed axial tomography (CAT or CT) [102,104]. In addition, further functional data from positron emission tomography (PET) [105,106], single photon emission computed tomography (SPECT) [107,108], and functional magnetic resonance imaging (fMRI) [101,106,108,109] can be integrated with MEG and EEG to achieve a more comprehensive characterization of brain sources.

To summarize, MEG and EEG directly measure neuronal activity with excellent temporal resolution. However, spatial localization is dependent on the non-unique inversion problem. Magnetic resonance imaging (MRI) and computerized tomography (CT) yield high-resolution spatial anatomical images. In addition, functional magnetic resonance imaging (fMRI), positron emission tomography (PET), and single-photon emission computed tomography (SPECT) provide three-dimensional functional characterization of brain activity in terms of metabolic and hemodynamic processes. Compared with MEG and EEG, fMRI, PET, and SPECT limitations arise from the long-time constants of metabolic and hemodynamic processes as well as the poorly defined relationship between them and neuronal processes.

### 4.2. MEG Signals

In this section, details about the origin of the MEG signals will be described. More information concerning the magnetic field generated by the cellular mechanism can be found elsewhere [2,110,111,112]. MEG fields are the result of currents in the brain, particularly in the cerebral cortex. The cortex is composed of well-aligned pyramidal cells consisting of dendrites, cell bodies, and axons, with approximately 10^5^–10^6^ cells per 10 mm of cortex [113]. The cells are connected to each other by nerve fibers, which are connected to dendrites and cell bodies by synapses. The human brain contains approximately 10^10^ neurons and 10^14^ synaptic connections.

The cell body can be considered a tubular volume surrounded by a membrane. The Na-K pump mechanism [110] creates an excess of K^+^ ions inside the cell and Na^+^ ions outside. The resulting concentration gradients and differences in membrane permeability for K+ and Na+ ions cause the positive ions to diffuse across the membrane. The electrical and diffusion forces compete (Nernst equation [110]), creating a negative equilibrium potential of about −70 mV within the cell. Stimulation of the cell (chemical, electrical, or mechanical) can cause a change in its transmembrane potential and lead to its depolarization (or hyperpolarization). As the cell is conductive, the shift in polarization induces an intracellular current and a return current outside the cell through the brain.

The transmembrane currents, J_i_, are the ‘impressed’ currents that drive passive volume currents in conducting tissues outside the membrane. These volume currents contribute to the magnetic field via a sum of terms over all conductivity discontinuities, such as cell membranes and the macroscopic volume of the brain. Summing the cell boundary terms is equivalent to summing dipole sources, which can be expressed as a current dipole density (J_c_). Macroscopically, J_i_ and J_c_ behave as an effective current source, i.e., the primary source of biomagnetic fields (J_P_ = J_i_ + J_c_). Since the membrane volume is small and corresponds to terms associated with macroscopic discontinuities (e.g., brain boundaries [114]), the contribution of Ji to the magnetic field is negligible, and the primary sources can be expressed as J_P_~J_c_. Magnetic imaging studies aim to determine J_P_ from measurements. Action potentials or axonal currents are usually not observable magnetically, resulting in magnetic fields with fast spatial decay.

**Figure 15:**
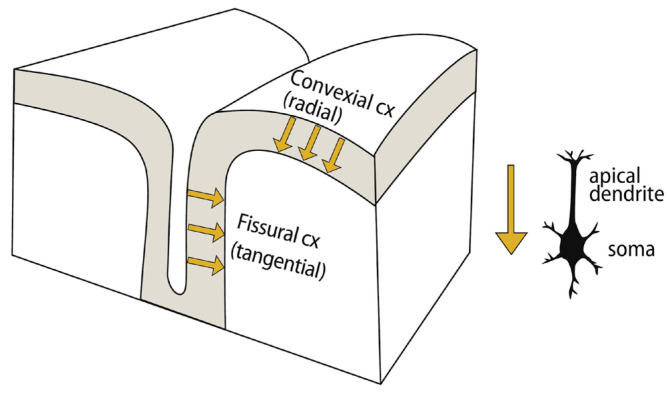
Schematic presentation of convexial and fissural currents in a slab of cortex, showing how these currents are formed and how they move. The central axis of pyramidal neurons is perpendicular to the cortical surface. These neurons are the primary sources of the MEG signals. Therefore, MEG signals are primarily generated by currents in the walls of fissures that are tangential to the surface of the skull (adapted from [115], an open-access article distributed under the terms of the Creative Commons Attribution License (CC BY).

MEG signals are often thought to arise from postsynaptic currents [2]. Postsynaptic dendritic currents flow roughly perpendicular to the cortex. However, the cortex has sulci and gyri, and the current flow is tangential depending on cell stimulation. As measured by MEG, radial magnetic fields are caused by tangential primary currents (see Figure 15).

The current within a cell is too small to produce observable fields outside the scalp. These fields are a result of many cells activating at the same time. MEG sources are usually distributed, but the activation of many cells is often small and can be modelled by a point equivalent current dipole (ECD). The current dipole density in brain tissue is nearly constant, ranging from 0.5 to 2 nA m/mm^2^. Typical ranges of magnetic field amplitudes encountered in MEG range from low 10 fT for the spinal cord to about 1 pT for α and δ bands, and in frequency from a fraction of a hertz to about 1 kHz. The brain field signals are many orders of magnitude smaller than environmental noise; the Earth’s magnetic field is about 10^−4^ T. Efficient noise cancellation is necessary because of the difference between the brain’s fields and the environmental noise magnitudes.

### 4.3. MEG System Description

MEG systems are complex installations involving various engineering disciplines. They involve SQUID or OPM sensors, cryogenics, noise cancellation, electronics, system design, software, data handling and interpretation, and neurosciences. SQUIDs or OPMs are necessary for MEG systems, but they represent only a small part of the overall system. Other important issues include noise cancellation, patient support, and head positioning.

SQUID sensors are mounted in liquid He dewars, which are usually suspended in a gantry for a supine or seated patient position. The dewar contribution to the noise could be about 1–2 fT/√Hz, limiting the sensitivity of shielded MEG systems. This noise is caused by thermal fluctuations in various conducting materials in the dewar vacuum space. Achieved noise levels of 0.08 fT/√Hz [116] have been reported for a dewar employing superinsulation with finely divided metallization and a vapor-cooled shield made from an electrically insulating ceramic.

**Figure 16:**
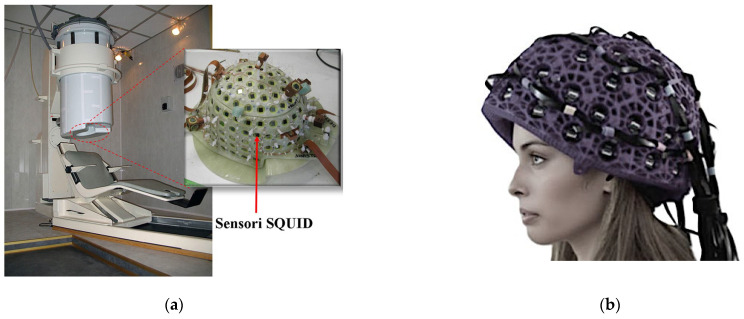
(**a**) Shielded room with an MEG system operating in an institute of diagnosis and treatment; the inset represents the helmet with the SQUID arrays (adapted from [121], an open-access article distributed under the terms of the Creative Commons Attribution License (CC BY)); (**b**) example of OPM helmet (adapted from https://nyaspubs.onlinelibrary.wiley.com/doi/10.1111/nyas.14935 (accessed on 25 May 2025), an open-access article distributed under the terms of the Creative Commons Attribution License (CC BY)).

The patient is seated on an adjustable chair or on a bed. The end of the dewar in contact with the patient is shaped like a helmet (Figure 16a). The inner dewar vessel is covered with the primary sensor, which could be magnetometers, radial gradiometers, planar gradiometers, or a combination. The helmet covers an area of approximately 0.1 to 0.12 m^2^ of the scalp. Even though the patient’s head is inside the MEG helmet, it can still move. This means that we need to know precisely where it is and how it faces the MEG array. This information then aligns the MEG results with the brain’s anatomy (for example, in MRI images). The most common and simplest head positioning method uses several easily identifiable anatomical landmarks. Special markers are placed at these points, and their positions are determined. However, this method is only accurate to a few millimeters due to compound placement errors of the MEG and MRI markers. Continuous head position monitoring using MEG sensors is also available [117,118]. To reduce registration problems, the head surface is digitized in the MEG system. This can be done by moving a small coil and detecting its position using MEG sensors or an electromagnetic tracking device [117]. The head surface is also determined from the MRI image, and the transformation between the systems is obtained by matching the surfaces of the two heads [119,120]. In the OPM-MEG system, the dewar and the refrigeration are unnecessary, and the helmet is more compact and portable, allowing movement during the brain scan, as reported in Figure 16b.

The SQUID system and patients are usually positioned in a shielded room. MEG rooms are typically shielded μ-metal rooms with an aluminum layer between two μ-metal layers. These can be obtained commercially from several manufacturers [122,123,124,125]. In Table 1, a comparison between the parameters of MEG based on SQUIDs and OPMs is reported.

EEG usually complements MEG measurements, and both signals are amplified and transmitted from the shielded room for further processing. MEG installations also have provisions for stimulus delivery and typically have an intercom and a video camera for observation and communication with the subject. Evoked MEG measurements use a range of stimulation equipment. Visual stimulators use nonmagnetic goggles, projector screens, or computer monitors. Sound stimulators usually deliver sound via nonmagnetic plastic tubing or to a shielded room via piping. Somatosensory experiments use electrical or tactile stimulators. Various switches and detectors are used for voluntary or forced finger movements.

Regardless of the method used, care must be taken not to introduce stimulation artefacts. The wires of electrical stimulators must be firmly twisted. Computer monitors must be tested for magnetic noise, and speakers must be placed far enough from the MEG system to avoid producing magnetic noise. MEG sensor noise is typically in the range of 3 to < 10 fT/√Hz for radial gradiometers or magnetometers [85,86] and about 0.3 fT/mm √Hz for planar gradiometers [85].

The vacuum gap in the dewar, typically 15 to 20 mm, separates the sensing coils from the scalp surface. Since the signal strength decreases with the square of distance from the source, the signal-to-noise ratio (SNR) and the resolution are limited by the gap between the source and the sensor. To reduce the limitation that arises from the distance between source and sensors, the OPM-MEG system offers the possibility to use a wearable helmet, which not only minimizes the distance dependence but allows more adaptability to a wider range of patients, without head dimension restriction. Moreover, if noise suppression references are present, they are located some distance from the primary sensors so that they detect ambient noise and are insensitive to brain signals.

Modern MEG investigations use whole-cortex helmet-type MEG instrumentation with many channels; systems with 64 and up to 306 channels have been produced [13,85,86]. These highly sensitive instruments use sophisticated array-based signal processing to enhance detection.

It is challenging to measure the magnetic fields of the human brain in the presence of high environmental noise. However, this technology is well understood, and MEG instruments are reliable and relatively straightforward. It is virtually impossible to examine all traces visually because modern instruments have many channels and generate enormous data. Various methods can be used to convert the collected data into a simpler presentation for a clinician or researcher. These techniques invert the magnetic fields around the scalp to reveal the brain’s electrical activity. However, the MEG inversion problem is non-unique and highly indeterminate, necessitating the introduction of various simplifying assumptions and constraints. This has led to a wide range of inversion methods. These differ in their assumptions about the brain and current sources and their mathematical details. The most common methods are the ECD, minimum norm, MUSIC, and beamformers [133].

## 5. MEG Clinical Applications

MEG is currently the most successful biomagnetism application. It is being used in research, presurgical mapping, and epilepsy. It is also used in research into pathological functional deficits, neuropharmacology, neuroscience, and psychiatry.

MEG provides information on brain activity at a millisecond scale, complementing functional imaging techniques such as fMRI and PET, which can determine the location of activity. Its older counterpart, the EEG, is also complemented by MEG. MEG detects the magnetic fields due to the primary and volume currents, and EEG detects their corresponding electric potentials on the scalp surface.

MEG and EEG must be acquired simultaneously to take advantage of their complementary information, and the electrodes must be non-magnetic to avoid MEG artefacts. If the EEG preamplifier impedance is greater than 1 MΩ, then the currents in the EEG electrode leads will not generate MEG artefacts. Both techniques measure the same current sources, which increases diagnostic accuracy.

In addition, fetal magnetoencephalography (fMEG) is a non-invasive technique used to study the developing brain of an unborn fetus by measuring the magnetic fields produced by electrical activity in the fetal brain. Currently, fMEG sensors are typically arranged in a helmet-like structure that surrounds the pregnant woman’s abdomen; this is the only non-invasive way to assess the neuronal activity of a fetus. It can measure fetal brain activity and assist physicians with high-risk pregnancy and diagnosis associated with infection, toxic insult, hypoxia, ischemia, and hemorrhage. The instrument can also evaluate fetal heart activity and other abdominal electrical activity.

Approximately 11% of people over 65 years old are affected by Alzheimer’s disease (AD), the most common form of dementia [134]. This neurodegenerative condition is associated with memory deficits and a decline in visuospatial and executive abilities. The pathophysiology of AD involves alterations in neural oscillations at low and high frequencies [135]. Resting-state neural oscillations are characterized by a decrease in spectral power at alpha, beta, and delta band frequencies as well as an increase in theta activity [136]. Symptom severity and cognitive deficits are linked to reduced alpha-band connectivity [137]. Task-related SQUID-MEG recordings show that alterations in neural oscillations affect memory networks [138], and the OPM-MEG system has measured oscillatory activity in theta band and gamma band [139,140]. However, SQUID-MEG has limitations due to the age and symptoms of AD patients; long periods of movement are hard to measure. By contrast, OPM-MEG systems have been shown to provide reliable data during movement, which could help with measurements on larger AD groups [141,142].

Schizophrenia is a severe psychiatric condition marked by impaired thinking and perception [143,144]. EEG and MEG research has investigated the causes of these deficits [145,146]. A consistent finding is reduced amplitude and synchrony of gamma-band (>30 Hz) oscillations in patients with schizophrenia [147,148]. These oscillations require two things: GABAergic interneuron-mediated inhibition and NMDA-receptor-mediated excitatory drive. Both have been implicated in circuit deficits in schizophrenia. Schizophrenia has also been linked to deficits in low-frequency rhythms, both during resting-state and task-related activity [149,150,151,152,153,154,155,156]. Superior signal-to-noise ratios are offered by OPM-MEG measurements in comparison to EEG [157,158] and possibly also to SQUID-MEG [118,129]. In addition, the evaluation of activity in the subcortical regions of the brain, such as the hippocampus and cerebellum, which have been implicated in Schizophrenia [159], can be very challenging using SQUID-MEG and EEG systems. Consequently, the enhanced flexibility provided by OPM-MEG systems could facilitate the measurement of these structures in patient populations [160,161].

Progressive motor symptoms characterize neurodegenerative movement disorders. These include tremors, rigidity, and abnormal postures. The course is often chronic and debilitating [162]. Examples of such disorders are Parkinson’s disease (PD) and dystonia. Researchers commonly observe aberrant oscillatory activity in the basal ganglia in these syndromes, primarily through intracerebral deep brain stimulation (DBS) electrodes [163,164,165,166]. This unique invasive approach has led to the idea that movement disorders should be considered network disorders. Specifically, oscillatory basal ganglia activity alterations indicate symptom patterns rather than disease-specific spectral patterns. Elevated beta oscillations (13–35 Hz) have been associated with hypokinetic symptoms, such as lethargy [167,168], while augmented low frequency (3–12 Hz) [169,170,171] and gamma activity (60–90 Hz) have been linked to hyperkinetic symptoms, such as involuntary movements or muscle contractions. Recordings at the network level have allowed us to study the patterns of brain activity during voluntary movements [172,173]. They have also revealed different pathways between the cortex and the basal ganglia in patients with PD [174,175].

Indeed, in patients with dystonia, simultaneous SQUID MEG and DBS recordings revealed a symptomatic decrease in alpha coherence (9–12 Hz) between the cerebellum and the internal pallidum, the basal ganglia output nucleus [176,177,178].

One significant challenge in this regard is the difficulty of investigating cerebellar activity using SQUID-MEG and EEG. OPM-MEG systems, however, have the potential to overcome this issue. Sensory symptoms, e.g., paresthesia, auditory impairments, and visual hallucinations, occur in >70% of patients with movement disorders. OPMs, placed flexibly at the lower back of the head close to the signal source, could improve recordings from sensory cortices, e.g., visual and deep auditory cortices [160]. This could significantly expand our understanding of the role of the sensory system in movement disorders. Moreover, the applicability and data quality of EEG and SQUID-MEG recordings are severely compromised by the limitations imposed by involuntary movements, particularly tremors. By contrast, OPMs can provide high-quality findings even during such movements.

Affecting more than 50 million people worldwide, epilepsy is one of the most common neurological disorders. Unfortunately, despite treatment with at least two adequately dosed antiepileptic drugs, one third of people with epilepsy continue to have seizures [179].

Some of these individuals will therefore require a more detailed assessment, including, in some cases, invasive EEG recordings, to identify the epileptic focus and to delineate further the eloquent cortical structures that need to be preserved. Many of these invasive approaches are associated with a significant risk of complications, in particular hemorrhage [180]. Interictal MEG is useful in providing new information in cases of drug-resistant epilepsy. A study of 1000 patients showed that MEG provided additional useful information in 32% of cases with focal onset seizures. This means that MEG can sometimes replace or complement presurgical non-invasive procedures. Overall, MEG use is associated with a higher rate of post-operative seizure freedom [181].

A first study applying OPM-MEG to children suffering from epilepsy showed that a 32-sensor system could detect interictal spikes with higher SNR and comparable localization accuracy compared to a 204-sensor SQUID-MEG system [141]. OPM-MEG may offer several advantages with respect to SQUID-MEG, such as a measurement during head movements and even locomotion during recordings [141], which is of particular importance for young children, the pediatric population, and individuals with intellectual deficit and/or behavioral problems [182].

**Figure 17:**
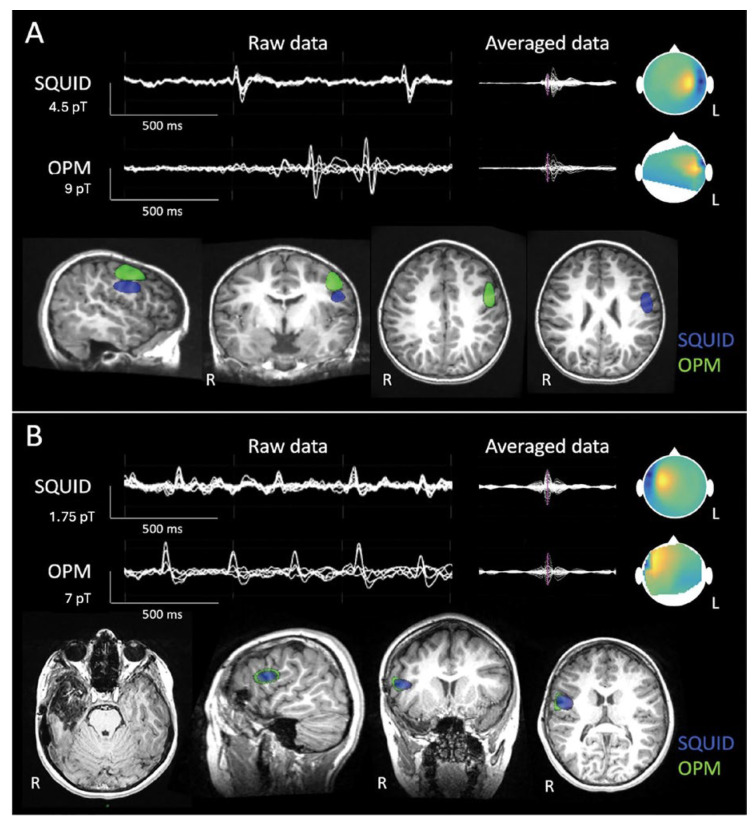
Encoding of epileptic spike obtained using OPM-MEG and SQUID-MEG. (**A**) Images in a 5-year-old girl (participant 3) with self-limited epilepsy with centrotemporal spikes. (**B**) Images in an 11-year-old girl (participant 5) with refractory focal epilepsy. Adapted from [141], an open-access article distributed under the terms of the Creative Commons Attribution License (CC BY).

Figure 17 presents a comparison between MEG traces with epileptic spikes measured by OPM- and SQUID-based MEG.

## 6. Conclusions and Perspectives

This review focuses mainly on the superconducting quantum magnetometers involved in one of the biomagnetic applications, magnetoencephalography (MEG). While biomagnetism has been detected in many organs, the field has been most extensively applied to the brain. MEG studies have gradually moved from research laboratories into clinical practice. This work reported a detailed description of the most extensive sensor used for MEG, the SQUID magnetometer. Today, MEG systems have also been developed using magnetometers based on detecting the Larmor spin precession of optically pumped atoms (i.e., Rb), called OPM. Their basic working principles and main advantages over the SQUID magnetometer, particularly in clinical applications where patient movement is unavoidable, were described. Moreover, a brief illustration of the brain signals detected by the MEG system and the MEG instrumentation components, such as cryogenic equipment, patient support, and a shielded room, was provided. An overview of MEG’s increasing and successful application in studying many degenerative diseases and brain illnesses was presented.

Finally, the success of MEG in providing a direct measure of neural activity with excellent temporal resolution has encouraged progress and interest in this technology. Using the MEG system in basic and clinical research could provide new insights into the origins and diagnosis of psychiatric and neurological disorders. Developing new quantum magnetometers for the MEG system could improve the performance of such diagnostic instruments, determining a much broader application in many other neuroscience fields.

## Figures and Tables

**Table 1:** Comparison between main parameters of SQUID-MEG AND OPM-MEG.

*Parameter*	*SQUID-MEG*	*OPM-MEG*	*Reference*
**Operating temperature**	Cryogenic cooling (4.5 K)	* T_int_ 150 °C; T_ex_ 40 °C	[126]
**Noise floor**	2–5 fT/√Hz	7–10 fT/√Hz	[127]
**Dynamic range**	± 20 nT	±5 nT (up to ±150 nT in closed loop)	[126]
**Bandwidth**	Up to MHz	Up to 2 kHz	[128]
**Field strength (source depth 4 cm)**	30 fT (3 cm from the scalp)	100 fT (6 mm from the scalp)	[129]
**Shielding**	required	required	[126]
**Spatial resolution**	millimeters	millimeters	[130]
**Distance from scalp**	2–3 cm	6 mm	[131,132]
**Helmet**	Rigid, movement restriction	Wearable no movement restriction	[131]
**Cost**	High maintenance	low maintenance	[132]

* T_int_ temperature inside the vapor cell, T_ex_ = temperature outside the sensor casing.
